# Clinical feasibility of a commercially available MRI‐only method for radiotherapy treatment planning of the brain

**DOI:** 10.1002/acm2.14044

**Published:** 2023-06-21

**Authors:** Iiro Ranta, Pauliina Wright, Sami Suilamo, Reko Kemppainen, Gerald Schubert, Mika Kapanen, Jani Keyriläinen

**Affiliations:** ^1^ Department of Physics and Astronomy University of Turku Turku Finland; ^2^ Department of Medical Physics Turku University Hospital Turku Finland; ^3^ Department of Oncology and Radiotherapy Turku University Hospital Turku Finland; ^4^ HUS Diagnostic Center University of Helsinki and Helsinki University Hospital Helsinki Finland; ^5^ Philips Clinical Science MR‐Therapy Best The Netherlands; ^6^ Department of Medical Physics Medical Imaging Center Tampere University Hospital Tampere Finland; ^7^ Department of Oncology Unit of Radiotherapy Tampere University Hospital Tampere Finland

**Keywords:** brain radiotherapy, MRI‐only, RTP

## Abstract

**Background:**

Advancements in deep‐learning based synthetic computed tomography (sCT) image conversion methods have enabled the development of magnetic resonance imaging (MRI)‐only based radiotherapy treatment planning (RTP) of the brain.

**Purpose:**

This study evaluates the clinical feasibility of a commercial, deep‐learning based MRI‐only RTP method with respect to dose calculation and patient positioning verification performance in RTP of the brain.

**Methods:**

Clinical validation of dose calculation accuracy was performed by a retrospective evaluation for 25 glioma and 25 brain metastasis patients. Dosimetric and image quality of the studied MRI‐only RTP method was evaluated by a direct comparison of the sCT‐based and computed tomography (CT)‐based external beam radiation therapy (EBRT) images and treatment plans. Patient positioning verification accuracy of sCT images was evaluated retrospectively for 10 glioma and 10 brain metastasis patients based on clinical cone‐beam computed tomography (CBCT) imaging.

**Results:**

An average mean dose difference of *D*_mean_ = 0.1% for planning target volume (PTV) and 0.6% for normal tissue (NT) structures were obtained for glioma patients. Respective results for brain metastasis patients were *D*_mean_ = 0.5% for PTVs and *D*_mean_=1.0% for NTs. Global three‐dimensional (3D) gamma pass rates using 2%/2 mm dose difference and distance‐to‐agreement (DTA) criterion were 98.0% for the glioma subgroup, and 95.2% for the brain metastasis subgroup using 1%/1 mm criterion. Mean distance differences of <1.0 mm were observed in all Cartesian directions between CT‐based and sCT‐based CBCT patient positioning in both subgroups.

**Conclusions:**

In terms of dose calculation and patient positioning accuracy, the studied MRI‐only method demonstrated its clinical feasibility for RTP of the brain. The results encourage the use of the studied method as part of a routine clinical workflow.

## 1 INTRODUCTION

State‐of‐the‐art radiotherapy planning (RTP) utilizes co‐registered computed tomography (CT) and magnetic resonance (MR) images.^1^ CT provides electron density information required for performing dose calculation, which is not directly available from MR data.^2^ The additional magnetic resonance imaging (MRI) provides excellent soft tissue contrast and tissue‐specific functional information. Compared with a CT‐based RTP, introduction of complementary MRI has improved the delineation accuracy of planning target volumes (PTV) and organs‐at‐risk (OAR),^3^, ^4^, ^5^ at the expense of requiring imaging with two modalities.

Despite being beneficial in gross tumor volume (GTV) and clinical target volume (CTV) delineation,^6^, ^7^ the co‐registration of CT and MR images also introduces uncertainties in the RTP process.^8^ Co‐registration uncertainties are caused by variations in patient positioning between both exams, and potential geometric distortions present in the MRI images.^1^ Selection of co‐registration method and image quality can also impact the quality of the co‐registration.^9^

Historically, geometric distortions in MR images have been reported to be significant on tissue interfaces and in the vicinity of air cavities.^10^ In the brain region, the magnitude of spatial uncertainties has been shown to be up to 2 mm.^8^, ^9^ This scale of uncertainty is even more significant in stereotactic radiotherapy of the brain, where the total planning uncertainties should remain well below 1 mm to ensure sufficient plan quality for the treatment of the smallest lesions.^11^ However, scanner hardware improvements and advancements in MRI sequence design have mitigated the scanner‐ and patient‐induced geometric distortions to enable the use of MRI in RTP.^12^

If in addition, the electron density information is derived from MR images through generation of synthetic CT (sCT) images, an MRI‐only‐based workflow for RTP can be implemented. The advantages of MRI‐only RTP workflow are minimization of the co‐registration uncertainties and improvement of resource and cost efficiency by omission of the CT imaging.^13^ Both conventional image‐guided radiotherapy (IGRT) and emerging radiotherapy (RT) techniques, such as MRI‐guided radiotherapy^14^ will benefit from the development in MRI‐only RTP methods, as they will be essential for instance in daily adaptive RTP.^15^

During recent years, different sCT conversion methods have been investigated and several types of approaches have been successfully adapted to generate sCT images capable of accurate dose calculation in the brain.^16^, ^17^ Previous studies have also indicated the feasibility of sCT‐based digitally reconstructed radiographs (DRR) for patient positioning verification compared with CT‐based patient positioning verification in radiotherapy (RT).^18^, ^19^, ^20^ Recently, the number of deep‐learning and artificial intelligence (AI)‐based sCT generation methods for MRI‐only RTP of the brain has been steadily increasing.^21^

Whenever new methods for RTP workflow are introduced, there is a need to clinically verify the performance of such methods before applying them into clinical practice, as the hardware and software configurations between individual units can vary. However, the investigation regarding the clinical feasibility of commercial MRI‐only solutions in the brain area has been limited, and to our knowledge, only two studies have evaluated the clinical feasibility of another commercial sCT generation method in MRI‐only RTP of the brain.^22^, ^23^

This study investigates the clinical feasibility of MRI‐only RTP of the brain on patients with glioma and brain metastases along with the associated differences in imaging protocols and treatment plans. A commercial, deep learning ‐based sCT generation method is evaluated with regard to dose calculation accuracy and patient positioning verification. The evaluations are performed using clinical tools only. Patient positioning verification in IGRT setting is evaluated using kilovoltage (kV) x‐ray cone‐beam computed tomography (CBCT).

## 2 METHODS

### 2.1 Patient cohort

For the comparison of dose calculation accuracy, 50 patients undergoing external beam radiotherapy (EBRT) of the brain in Turku University Hospital (Turku, Finland) were retrospectively selected for the current study. The patient cohort was divided into glioma and brain metastasis patient subgroups, each consisting of 25 patients. The glioma patients were planned with using volumetric modulated arc therapy and metastasis patients using conformal arc stereotactic radiotherapy techniques and 6 MV flattened and flattening filter free beam photons, respectively. Clinical patient cohort details and structure volume information are presented in Table 1.

**TABLE 1:** Patient cohort details including the PTV and NT volumes for the evaluation of dose calculation accuracy.

Prescribed dose	Glioma Range (Gy)	Metastasis
	36.0–60.0	18.0–35.0

Structure	Mean volume (cm^3^) (SD) [Range]	
PTV	331.7 (168.4)	12.2 (12.7)
	[20.9–821.3]	[0.1–54.6]
NT	671.3 (219.5)	150.6 (66.0)
	[214.6–1152.7]	[53.5–330.2]

Abbreviations: NT, normal tissue, definition described in Section 2.6; PTV, planning target volume; SD, standard deviation.

### 2.2 Imaging

CT imaging was performed with a Toshiba Aquillion LB (Toshiba Corp., Tokyo, Japan) using the default CT simulation protocol of 120 kV tube voltage, 50 mA tube current, and 1.0 × 1.0 mm^2^ reconstruction resolution using iterative reconstruction. CT images were acquired with a slice thickness of 1.0 mm. The image reconstruction slice thickness for brain metastasis patients was 1.0 and 2.0 mm for glioma patients.

MRI was performed with a Philips Ingenia 1.5 T MR‐RT scanner (Koninklijke Philips N.V., Best, The Netherlands). A T1‐weighted 3D mDIXON imaging sequence was used to collect the source MRI data for the sCT conversion. The imaging sequence parameters are presented in Table 2.

**TABLE 2:** Source MRI imaging parameters.

Sequence	Acq. voxel size (mm^3^)	Recon. voxel size (mm^3^)	TE1/TE2 (ms)	TR (ms)	Flip angle (°)	WFS (px)	Scan time (min:s)
T1 3D FFE mDIXON	1.1 × 1.1 × 1.4	0.68 × 0.68 × 1.0	2.0/4.4	6.8	20	0.452	2:56

Abbreviations: BW, bandwidth; MRI, magnetic resonance imaging; px, pixel; TE, echo time; TR, repetition time; WFS, water fat shift.

Both CT and MRI were acquired with thermoplastic head fixation (Orfit Industries N.V., Wijnegem, Belgium). Since the use of a diagnostic head coil was not possible with the used fixation equipment, the MRI signal data were acquired using two‐round, single‐channel flex coils positioned laterally on both sides of the head and the scanner‐integrated posterior coil.

### 2.3 sCT image generation

The sCT images were generated using a commercially available, deep‐learning based algorithm (magnetic resonance for calculating attenuation, MRCAT Brain, version 4.0; Philips Oy, Vantaa, Finland). The proprietary algorithm uses the MRI data from a fixed source scan and inputs it into a fixed convolutional neural network trained using matching pairs of CT and source MRI images. The algorithm uses continuous Hounsfield unit (HU) to electron density calibration curve for sCT image conversion. The sCT images are generated automatically on the scanner console as an image post‐processing step.

### 2.4 Quantitative image quality evaluation

Image quality of the sCT images was compared with corresponding CT images and evaluated by determining the mean absolute error (MAE) and mean error (ME) of HU values averaged over the volume within the body outline contour. The evaluations were performed with MATLAB (MATLAB 2015b; The MathWorks Inc., Natick, Massachusetts, USA) software using dedicated scripts. In order to enable good comparability between the clinical CT and sCT image data, the sCT images were first rigidly co‐registered with the CT images and then resampled to the same image grid using b‐spline transformation interpolation with six degrees of freedom. The six degrees of freedom are the translations in three dimensions, that is, left‐right, anterior‐posterior, and cranio‐caudal directions and rotations on three axes, that is, pitch, roll, yaw, respectively.

### 2.5 Evaluation of dose calculation accuracy

The dose calculation accuracy was assessed using Eclipse (version 15.6; Varian Medical Systems Finland Oy, Helsinki, Finland) treatment planning system (TPS). The sCT image sets were imported to the TPS and rigidly co‐registered with the existing CT images with six degrees of freedom. Clinical CT‐based RT plans were then recalculated using the sCT images as the base attenuation data. Eclipse's anisotropic analytical algorithm (AAA, version 15.6.04) was used to perform all dose calculations. The dose calculation grid size for glioma patients was 2.0 × 2.0 × 2.0 mm^3^and 1.0 × 1.0 × 1.0 mm^3^ for brain metastasis patients. While the CT‐sCT co‐registration was performed using six degrees of freedom, due to limitations of the TPS, these rotations were not propagated during the dose recalculation step. Therefore, rotational discrepancies between the CT‐ and sCT‐based RT plans affected the dose calculation accuracy results. With this approach exclusively based on clinical tools, the aim was to demonstrate a scalable solution for other RT units, and to point out the potential consequences for the results.

Dosimetric comparison of the clinically used treatment plans was performed based on the dose volume histogram (DVH) data for the PTV and normal tissue (NT) structures. Due to the variation in PTV location and volume, the NT structures within the body outline were created by adding a 2 cm outer margin to the PTV and then subtracting the PTV to improve comparability between patients. The NT structure was clipped at the body outline but extended into bone and inner air cavities. The DVH parameters *D*_max,_ *D*_2,_ *D*_50_ *D*_95_ *D*_98_, and *D*_mean_ for the PTVs were selected according to ICRU 83 report guidelines.^24^ Equivalent DVH parameters were used to obtain the NT results. The dosimetric evaluation for all structures was performed by calculating local relative dose differences between the DVH points with the following equation:

(1) $$\Delta D\left(V\right)\;=\frac{D_{\mathrm{sCT}}\left(V\right)-D_{\mathrm{CT}}\left(V\right)}{D_{\mathrm{CT}}\left(V\right)}\;$$

where ∆*D*(*V*) is the local relative difference of a dose point on the DVH curve, and *D*_CT_(*V*) and *D*_sCT_(*V*) are the calculated doses for the corresponding volume *V* in the CT and sCT‐based RT plans, respectively. Then, *V* represents the volume of a structure receiving a dose greater than or equal to dose *D*_CT_ or *D*_sCT_.

In addition to direct dosimetric comparison, the equivalence of CT and sCT‐based RT plans was evaluated by performing a 3D gamma analysis across the head volume with 1%/1 mm, 2%/2 mm, and 3%/3 mm dose difference and distance‐to‐agreement (DTA) criteria. The gamma analyses were performed using Slicer (version 4.11.20210226, slicer.org)^25^ open‐source software together with SlicerRT (version 1.0.0, slicerrt.github.io) extension.^26^ Dose threshold >10% of the maximum dose, default maximum gamma value of two, and geometric gamma calculation option^27^ were used to determine the gamma pass rates.

#### 2.5.1 The impact of rotational discrepancies

In order to illustrate the impact of rotational discrepancies, complementary resampling of the sCT images was done for two patients with worst outlier gamma acceptance rates (based on the results of 1%/1 mm gamma criterion for a brain metastasis patient and 2%/2 mm gamma criterion for a glioma patient). The sCT images were resampled to the same grid with the CT images using linear transformation interpolation. The dose calculation accuracy evaluation including the parametric DVH comparison and gamma evaluation was then repeated for the resampled sCT images and CT images as described in Section 2.5.

### 2.6 Patient positioning verification imaging

To evaluate the patient positioning verification accuracy of sCT images compared with CT images, an additional cohort of 10 glioma and 10 metastasis patients not included in the dose calculation accuracy evaluation were selected for a retrospective evaluation. Patient positioning verification was performed according to clinical routine with CBCT (100 kV tube voltage, 75 mAs exposure, 0.5 × 0.5 mm^2^ reconstruction resolution, and 2.0 mm slice thickness) using the imager system of Varian TrueBeam linear accelerators.

### 2.7 Accuracy of CBCT patient positioning verification

Evaluation between CBCT to CT‐ and CBCT to sCT‐based patient positioning accuracy was performed in the image registration workspace of Varian Eclipse. First, the planning sCT images were co‐registered to planning CT images according to skull bone anatomy, using a rigid registration with six degrees of freedom. The registration was performed using the auto‐matching feature of Eclipse with a downhill simplex optimization method and mutual information similarity measure options enabled without any additional filters and a tolerance value of 0.001.

The co‐registration process was performed similarly for the clinical CBCT images, which were co‐registered with both CT and sCT images. By using the resulting registration matrix information, the difference between CT‐ and sCT‐based CBCT registrations could then be estimated for each patient by subtracting the sCT‐based registration matrix from the respective CT matrix.

### 2.8 Statistical analysis

The statistical significance in MAE, ME, dose calculation accuracy, and gamma analysis results between the subgroups was evaluated with two‐sample *t*‐test for paired samples. The significance of positioning verification accuracy between subgroups was assessed using Wilcoxon signed‐rank test, to assess the differences in image resolution, PTV size and location, and treatment technique between the subgroups. A significance level of *p* = 0.05 was used in all statistical tests. JMP (version 16; SAS Institute Inc., Cary, North Carolina, USA, 1989–2021.) was used to perform all statistical analyses.

## 3 RESULTS

**FIGURE 1:**
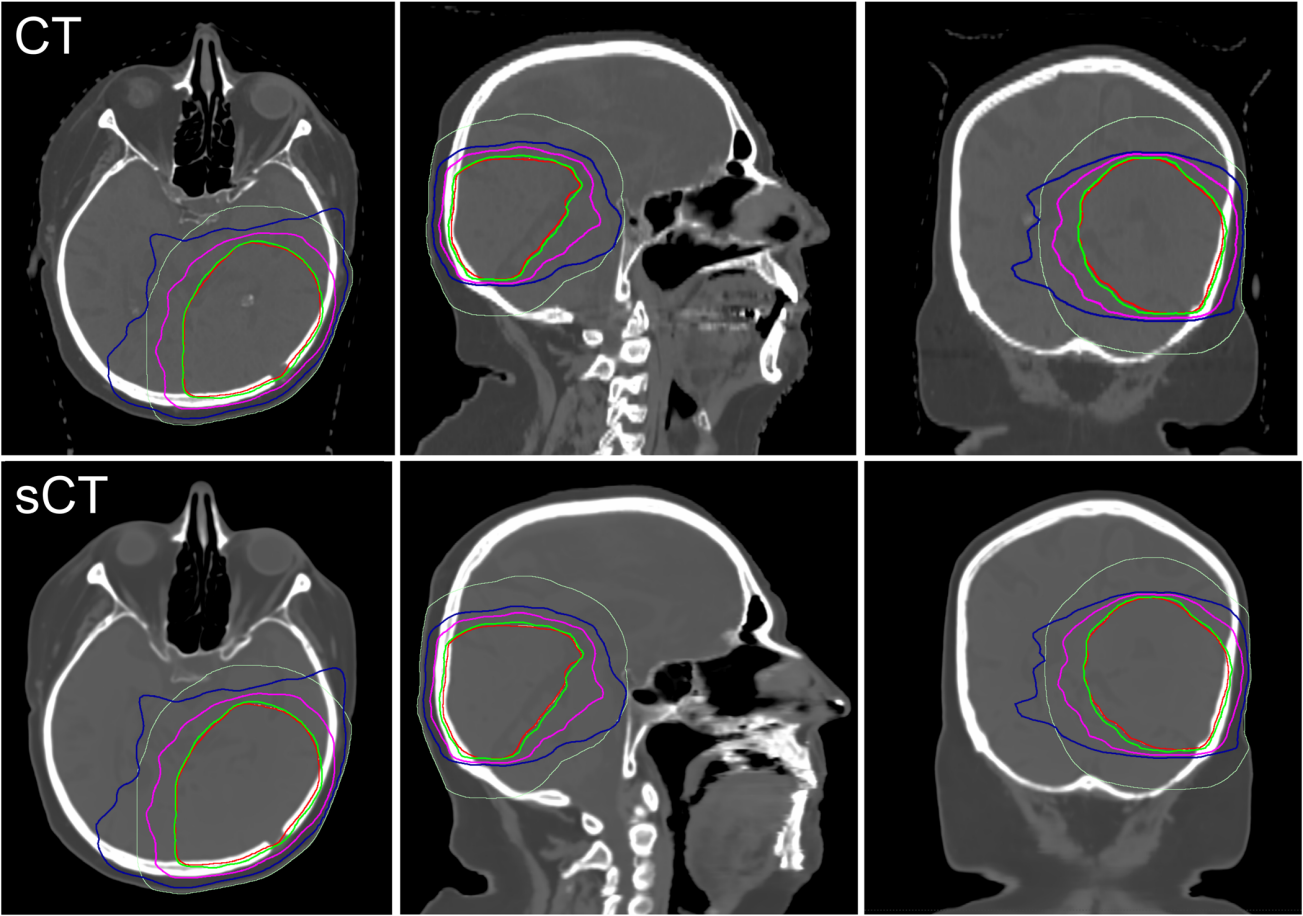
A case example of CT versus sCT image quality of a glioma patient with identical windowing parameters, showing the PTV (red) and the 2 cm NT (light green) structure outlines. Relative isodose contours of 95% (green), 70% (magenta), and 50% (blue) are visible. CT, computed tomography; NT, normal tissue; PTV, planning target volume; sCT, synthetic computed tomography.

The sCT images were successfully generated and sCT‐based RT plans calculated for all 50 patients. An example of generated sCT image quality and sCT‐based RT plan quality dose distributions compared with the corresponding CT image and CT‐based RT plan quality is presented in Figure 1. An example of plan quality for worst‐case glioma patient is presented in Figure A1.

### 3.1 Quantitative image quality evaluation

**FIGURE 2:**
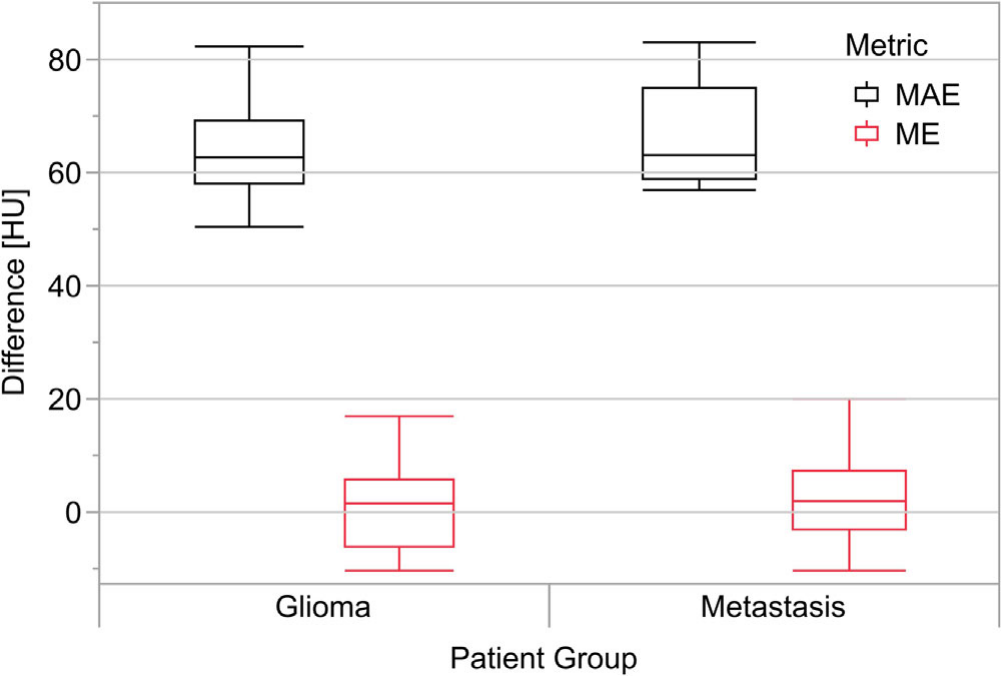
The HU comparison results showing box plots for MAE and ME metrics in glioma and brain metastasis groups. The box indicates the values between 25% and 75% quartile of the distribution, that is, the interquartile range (IQR), while the middle line depicts the median. The whiskers extend up to 1.5 times the IQR from the box borders, or to the extreme data point, whichever is closer. HU, Hounsfield unit; MAE, mean absolute error; ME, mean error.

**FIGURE 3:**
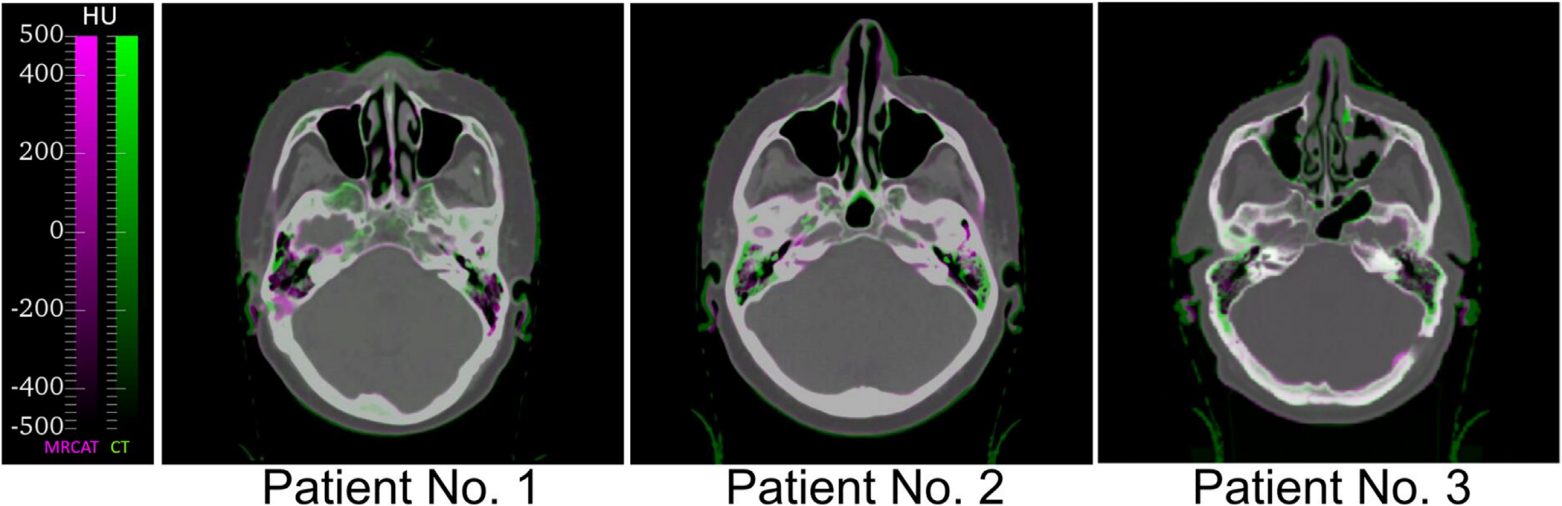
Three example images displaying the worst‐case differences of HU values between sCT and CT images. The images are displayed in green (CT) ‐ magenta (sCT) overlay. Voxels with matching HU values in CT and MRCAT images have a grey hue of the corresponding intensity. The majority of differences can be observed in areas with air cavities and cortical bone structures. CT, computed tomography; HU, Hounsfield unit; sCT, synthetic computed tomography.

Pooled HU comparison results for glioma and brain metastasis subgroups are presented in Figure 2 and in Table A1 of Appendix. An example of HU difference in different regions of the head is illustrated in Figure 3. No statistically significant differences between the subgroups were observed for MAE values.

### 3.2 Dosimetric comparison

**FIGURE 4:**
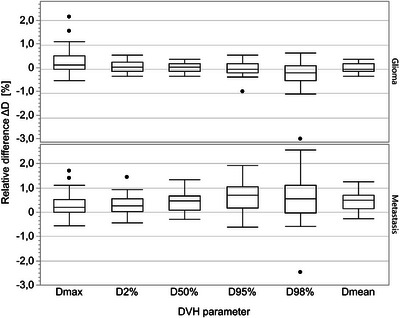
DVH comparison results for glioma and brain metastasis groups showing box plots for PTV structures. The box indicates the values between 25% and 75% quartile of the distribution, that is, the IQR, while the middle line depicts the median. The whiskers extend up to 1.5 times the IQR from the box borders, or to the extreme data point, whichever is closer. The outliers are represented as dots. D, dose; DVH, dose volume histogram; IQR, interquartile range; PTV, planning target volume.

Mean relative dose differences according to Equation (1) for all dosimetric parameters of the PTV were found to be ≤0.6% with a standard deviation of 1.0%) in the entire patient cohort when evaluating the non‐resampled sCT images. For the NT DVH parameters, a mean relative dose difference of ≤1.7% (3.6%) was determined. Statistical testing showed significant dose differences in PTV dose calculation accuracy results between patient subgroups (*p* < 0.05) for all except *D*_max_ DVH point (*p* = 0.80). For NT DVH parameters, there were no statistical differences between subgroups. The results for predefined dose calculation accuracy parameters are presented in Figure 4. Numerical data of both PTV and NT results are presented in Table A2 of Appendix.

**FIGURE 5:**
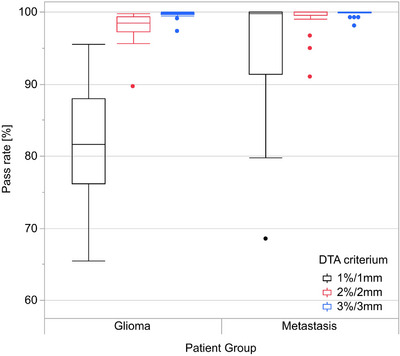
Box plots of gamma analysis results for glioma and brain metastasis groups with all predetermined dose differences and DTA criteria included. The box indicates the values between 25% and 75% quartile of the distribution, that is, the IQR, while the middle line depicts the median. The whiskers extend up to 1.5 times the IQR from the box borders, or to the extreme data point, whichever is closer. The outliers are represented as dots. DTA, distance to agreement; IQR, interquartile range.

Gamma analysis results yielded 98.0% (2.1%) pass rate when using the 2%/2 mm DTA criterion for glioma patients and 99.2% (2.0%) pass rate for brain metastasis patients. The stricter 1%/1 mm DTA criterion yielded pass rate of 95.2% (8.5%) for metastasis patients and 82.1% (7.6 %) for glioma patients. In total, one outlier patient in glioma and one outlier patient in metastasis subgroup was found with pass rate <95% when using the 2%/2 mm DTA criterion. Gamma analysis results are presented in Figure 5. Numerical results of the gamma analysis are presented in Table A3 of Appendix.

#### 3.2.1 The impact of resampling

**FIGURE 6:**
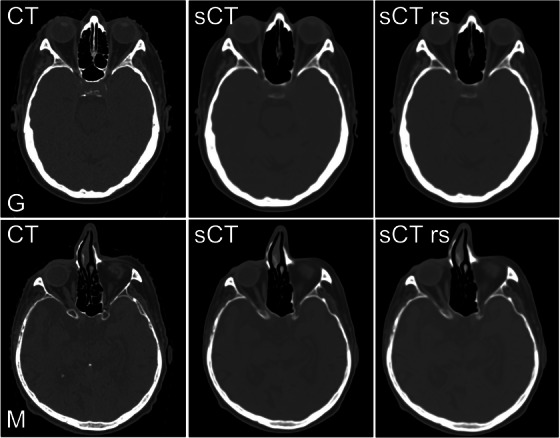
Image quality comparison between the CT, sCT, and resampled sCT (sCT rs) images used during resampling effect evaluation. Images for the outlier glioma patient are shown in the upper row. Images for the outlier metastasis are shown in the bottom row. Images are presented with identical windowing settings. CT, computed tomography; G, glioma; M, metastasis; rs, resampled; sCT, synthetic computed tomography.

The resampling was done for the worst case outlier glioma and brain metastasis patients. The image quality comparison between CT, sCT, and resampled sCT images is presented in Figure 6. Resampling of the sCT images for the re‐evaluated patients resulted in significant increase in gamma pass rates to >95% level. Also, the parametric DVH comparison results showed significant differences compared to non‐resampled sCT results. The results of the non‐resampled and resampled sCT plans compared with the CT plans are shown in Table 3. A detailed comparison of the HU value and dosimetric differences and gamma maps for the analyzed glioma and metastasis patients are presented in Figure A2 and Figure A3 of Appendix.

**TABLE 3:** Comparison of non‐resampled and resampled: (a) PTV DVH comparison results and (b) gamma pass rate results for the worst outlier glioma and brain metastasis patients.

(a)		
	Non‐resampled	Resampled
**Gliom**	**Relative dose difference to CT plans (%)**	
Δ*D*_max_	0.2	0.1
ΔD_2_	0.1	0.1
ΔD_50_	0.2	0.2
ΔD_95_	‐0.1	0.1
ΔD_98_	‐0.5	0.2
Δ*D*_mean_	0.0	0.2
**Metastasis**	**Relative dose difference to CT plans (%)**	
Δ*D*_max_	1.7	1.8
ΔD_2_	0.6	0.5
ΔD_50_	0.7	0.5
ΔD_95_	‐0.6	0.6
ΔD_98_	‐2.5	0.6
Δ*D*_mean_	0.4	0.6

(b)		
	Non‐resampled	Resampled
**Glioma**	**Pass rate (%)**	
1%/1 mm	65.5	96.2
2%/2 mm	97.4	99.4
3%/3 mm	97.4	99.8
**Metastasis**	**Pass rate (%)**	
1%/1 mm	68.6	99.3
2%/2 mm	91.0	99.8
3%/3 mm	98.1	100.0

Abbreviations: CT, computed tomography; D, dose; DVH, dose‐volume histogram; PTV, planning target volume.

### 3.3 Patient positioning verification

**FIGURE 7:**
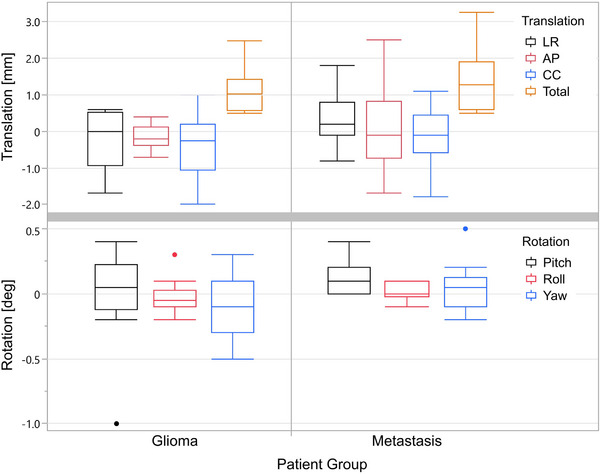
Box plots of patient positioning verification results for glioma and brain metastasis groups. The box indicates the values between 25% and 75% quartile of the distribution, that is, the IQR, while the middle line depicts the median. The whiskers extend up to 1.5 times the IQR from the box borders, or to the extreme data point, whichever is closer. The outliers are represented as dots. AP, anterior‐posterior; CC, cranio‐caudal; deg, degrees; IQR, interquartile range; LR, left‐right.

The results for accuracy assessment of patient positioning verification for CBCT images presented in Figure 7 and Table A4 of Appendix showed on average less than 1.0 mm difference in primary coordinate directions between CT and sCT‐based positioning in both subgroups. The mean rotational difference in all axes of freedom was ≤0.1˚ in both subgroups. Statistical evaluation indicated statistically significant difference only for roll (*p* = 0.03); however, due to the absolute difference of 0.05˚, this had no clinical impact.

## 4 Discussion

The aim of this study was to evaluate the clinical feasibility of a commercial MRI‐only method for RTP of the brain in terms of dose calculation and patient positioning accuracy using clinically available tools. Overall, clinically significant dosimetric discrepancies, or differences in patient positioning were not found in the pooled data, that is, dosimetric differences between plans were below 2% in the PTV region. Similar criteria for clinical feasibility have been previously proposed by Korsholm et al. ^28^ Also, the gamma acceptance rate of over 95% was observed when using 2%/2 mm dose and DTA criteria.

The HU value comparison between CT and sCT images showed good agreement with previous research, which have reported similar MAE values.^22^, ^23^, ^29^, ^30^, ^31^ As illustrated in the examples in Figure 3, the largest HU differences are observed in the vicinity of air cavities and in regions with fine bone structures or body outline differences. While the results of dose calculation accuracy depend on the used HU calibration curves, the local uncertainties in image quality, such as tissue misclassifications, have a larger effect in the dose calculation accuracy. The expected difference between plans calculated using slightly different calibration curves should therefore be rather small, as demonstrated in previous studies comparing CT‐based plans.^32^, ^33^

The mean results of the dosimetric accuracy evaluation were coherent with previous feasibility research on glioma and brain metastasis patients using different sCT image generation methods.^16^, ^20^, ^22^, ^23^, ^34^ The largest relative dosimetric differences were observed in patients who had PTVs and therefore NTs extending into head air cavities. The occurrence of this type of outlier results can be expected, and it is in line with challenges faced also by other sCT algorithms and are due to absence of MR signal in the vicinity in both air and cortical bone as well as patient‐induced distortions in the vicinity of high susceptibility differences.^35^, ^36^ The use of a deep learning‐based generation method of the sCTs in MRCAT brain does not guarantee a perfect anatomical match in the most challenging anatomical sites of the head, for example, nasopharynx, oropharynx, and ear canals. In these types of challenging locations, the tendency for the algorithm was to overestimate the size of air cavities in sCT images compared with CT images.

The single largest outlier in the DVH comparison was observed for NT, the Δ*D*_95_ being 16.3%. This deviation was caused by the large volume NT structure covering areas very close to the skin surface, and partially extending into the oropharyngeal cavity. Both the body outline differences, and cortical bone regions had a pronounced impact on dose calculation results. However, the absolute ΔD_95_ was small (3.4 Gy in CT vs. 2.9 Gy in sCT) compared with prescribed dose (39 Gy) in the outlier case, which resulted in large relative dose difference with little clinical impact. For the same patient, the Δ*D*_mean_ in the NT region was 1.1% and Δ*D*_max_ was ‐0.1%.

Overall, higher differences of NT DVH parameters were caused by the body outline differences in combination with evaluating DVH metrics in regions with pronounced dose gradients or the rotational discrepancies between the CT and sCT images. In these regions, light geometric shifts of the spatial dose distribution have a pronounced effect on the DVH parameters compared with regions with a more gradual dose profile.

From a clinical perspective, it is important to take note of the limitations for dose calculation accuracy and dose optimization of each MRI‐only RTP method. When the limitations of the method are known, patient inclusion criteria for MRI‐only RTP workflow can be adjusted. While perfect correspondence between the gold standard CT and sCT plans is difficult to achieve, based on the results of the current work, the majority of patients with intra‐cranial lesions could be treated using an MRI‐only workflow in the brain area.

Gamma analysis between the CT and sCT‐based also yielded good results with mean pass rate of ≥95% when using the 2%/2 mm DTA criterion for glioma patients and 1%/1 mm DTA criterion for the brain metastasis patients. Similar results have also been reported in previous research on the brain area.^16^ Although there was a statistically significant difference between glioma and brain metastasis subgroups, the dosimetric accuracy for each subgroup was clinically sufficient. While the 1%/1 mm results for glioma patients are reported for completeness, it is important to note that the DTA component of the gamma criterion is below the resolution of the dose grid of 2 mm^3^. As brain metastases in general are substantially smaller in volume compared with gliomas, with spherical diameter of 2 mm in some cases, reporting the 2%/2 mm gamma pass rates provide little additional information about the sCT‐based plan quality of these targets.

The difference between subgroups in gamma analysis can be expected and they result from generally larger PTVs for glioma patients. A stricter gamma criterion for the brain metastases cases makes clinically sense in view of the smaller PTV size and steeper dose gradients in those cases. Also, the effect of rotational discrepancy between the non‐resampled sCT and CT images becomes more pronounced on extended distances from the treatment isocenter.

Overall, the rotational discrepancies between the non‐resampled sCT images and CT images were a significant source of uncertainty in the dosimetric evaluation of outlier patients. Based on the CT and sCT co‐registration matrices, the maximum rotations along any axis ranged from ‐2.4˚ to 2.1˚ for these patients. The impact of sCT image resampling was clearly demonstrated on both re‐evaluated patients. After the resampling step, the dosimetric disagreement could be seen to primarily result from body outline differences and possible tissue misclassifications around the air cavities. Also, gamma agreement was improved to over 95% after resampling even when using the stricter 1%/1 mm dose and DTA criterion.

When using clinical tools for dosimetric comparisons, it is important to recognize the effect of rotational discrepancies to dosimetric results and differentiate their effects from purely dosimetric differences between the CT and sCT‐based RT plans. In addition to resampling, the effects of both rotational discrepancies and body outline differences could have been alleviated by re‐optimization of the sCT RT plans as suggested by Paradis et al.^34^ However, this was not in the scope of the current clinical study. Despite the limitations of using non‐resampled sCT images for dosimetric evaluation, the clinical feasibility of the studied sCT generation method could be deemed sufficient.

The patient positioning verification results using a CBCT imaging approach showed that the positioning uncertainty when using sCT images as a reference was not clinically significant compared with normal variation between treatment fractions. Sub‐millimeter differences have also been reported in earlier research investigating CBCT‐sCT patient positioning^22^, ^31^.

Clinical MRI‐only RT of the brain is still a relatively recent advancement and not yet widely adapted in routine clinical workflows. This work investigated the compatibility of this approach for a single choice of equipment and workflow. Additional research should therefore be performed to clinically validate the patient positioning accuracy of sCT images when different patient positioning protocols, such as surface guidance or stereotactic imaging systems, are used. This could enable clinical MRI‐only workflow for a broader range of patients.

## 5 CONCLUSION

This study demonstrates the clinical feasibility of a commercially available MRI‐only method for the RTP of the brain and gives evidence of its implementation as part of a routine clinical practice for patients with glioma or brain metastases.

## AUTHOR CONTRIBUTIONS

The authors confirm contribution to the paper as follows: Conceptualization, Jani Keyriläinen, Mika Kapanen, Iiro Ranta, Pauliina Wright, Sami Suilamo, and Gerald Schubert; methodology, Jani Keyriläinen, Mika Kapanen, Iiro Ranta, Pauliina Wright, Sami Suilamo, and Gerald Schubert; software, Reko Kemppainen and Iiro Ranta; validation, Iiro Ranta, Pauliina Wright, and Sami Suilamo; formal analysis, Reko Kemppainen and Iiro Ranta; investigation, Iiro Ranta, Pauliina Wright, and Sami Suilamo; resources, Reko Kemppainen and Iiro Ranta; data curation, Iiro Ranta; writing—original draft preparation, Iiro Ranta; writing—review and editing, Jani Keyriläinen, Mika Kapanen, Iiro Ranta, Pauliina Wright, Sami Suilamo, and Gerald Schubert, and Reko Kemppainen; visualization, Iiro Ranta; supervision, Jani Keyriläinen, Mika Kapanen; project administration, Jani Keyriläinen, Mika Kapanen; funding acquisition, Jani Keyriläinen. All authors have read and agreed to the published version of the manuscript.

## CONFLICT OF INTEREST STATEMENT

Turku University Hospital and Philips Oy currently have a research agreement regarding the development of MRI‐only based techniques. Gerald Schubert is currently employed by Philips.

## ETHICAL CONSENT STATEMENT

The study was approved by the Ethical Committee of the Hospital District of Southwest Finland (reference code: Dnro 116/1801/2017, approval date: 21 November 2017, renewal date: 2 November 2020).

## Supporting information

Supporting Information
