# Supplementary material for: Clinical feasibility of a commercially available MRI‐only method for radiotherapy treatment planning of the brain
Source: J Appl Clin Med Phys. 2023 Jun 21;24(9):e14044. doi: 10.1002/acm2.14044 (PMC10476982; doi:10.1002/acm2.14044)
Supplement: Supplementary file 1 — Supporting Information [file ACM2-24-e14044-s001.docx]

Appendix

**
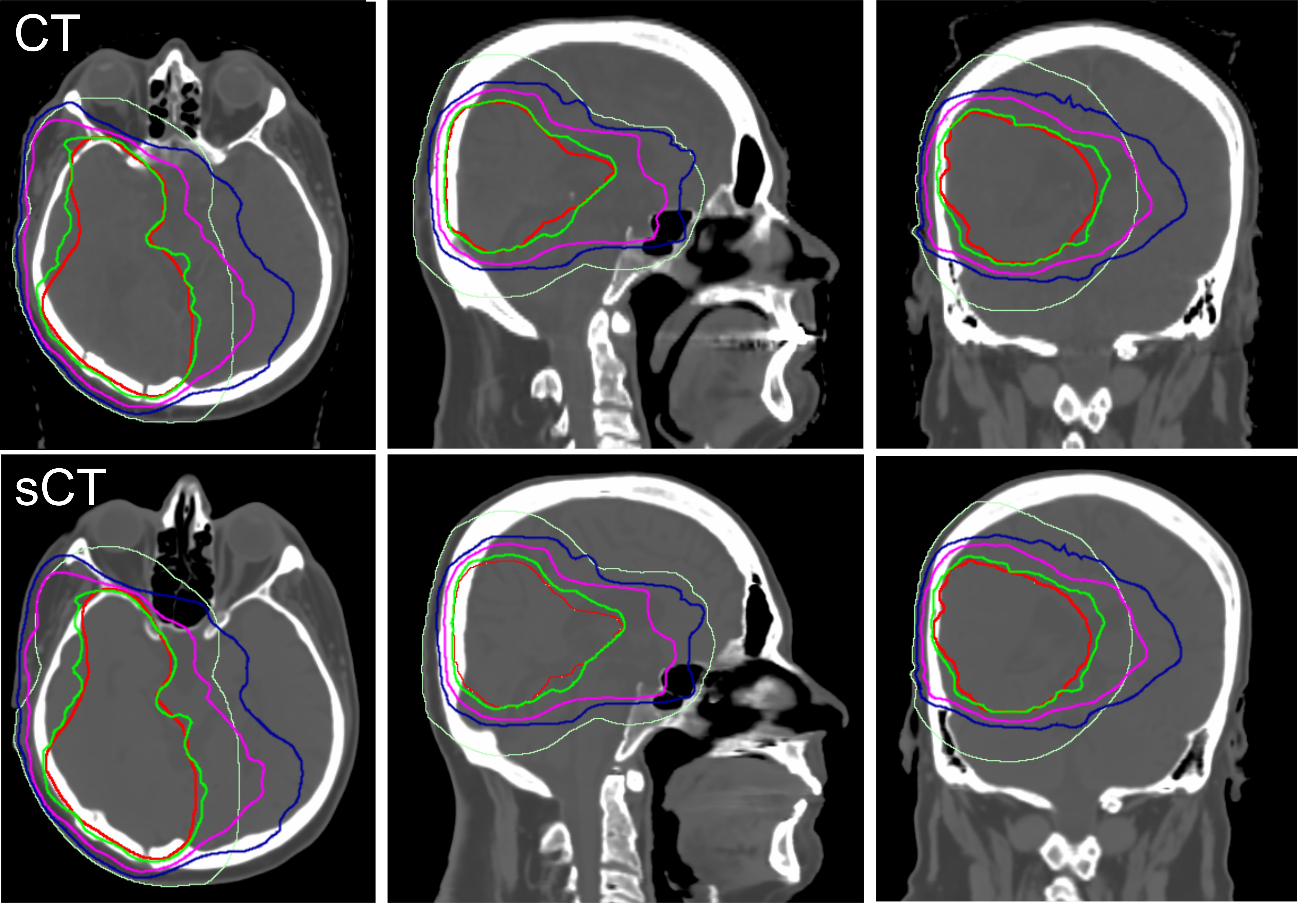
 Appendix Figure 1.** A case example of CT versus sCT image quality of the worst-case glioma patient with identical windowing parameters, showing the PTV (red) and the 2cm NT (light green) structure outlines. Relative isodose contours of 95% (green), 70% (magenta) and 50% (blue) are visible. (CT: computed tomography, sCT: synthetic computed tomography, PTV: Planning target volume, NT: normal tissue)

**Appendix Table 1.** The results of Hounsfield unit (HU) mean absolute error (MAE) and mean error (ME) evaluation for glioma and brain metastasis groups. (SD: standard deviation)

| Group | Glioma | Metastasis |  |
| --- | --- | --- | --- |
| Parameter | **Mean (SD) [Range] [HU]** | | **p** |
| MAE | 63.6 (8.7)  [50.4 – 82.1] | 66.8 (8.6)  [57.0 – 83.2] | 0.23 |
| ME | 1.1 (8.1)  [-10.5 – 17.0] | 2.1 (7.5)  [-10.6 – 20.0] | 0.67 |

**Appendix Table 2.**  Parametric dose calculation accuracy and results for glioma and brain metastasis patient subgroups. D_max_ is defined as the point maximum dose, D_2_ is the dose received by 2% of the total structure volume, etc. (D: Dose, DVH: dose-volume histogram, NT: Normal tissues, SD: standard deviation, PTV: planning target volume)

| Structure | PTV | |  | NT | |  |
| --- | --- | --- | --- | --- | --- | --- |
| Group | **Glioma** | **Metastasis** |  | **Glioma** | **Metastasis** |  |
| DVH parameter | **Mean relative difference (SD) [Range] [%]** | | **p** | **Mean relative difference (SD) [Range] [%]** | | **p** |
| ΔD_max_ | 0.3 (0.6)  [-0.5 – 2.2] | 0.3 (0.5)  [-0.6 – 1.7] | 0.80 | 0.3 (0.9)  [-2.0 – 2.2] | 0.8 (2.0)  [-2.0 – 8.5] | 0.90 |
| ΔD_2_ | 0.1 (0.2)  [-0.3 – 0.6] | 0.3 (0.4)  [-0.4 – 1.4] | 0.049 | 0.3 (0.3)  [-0.2 – 0.9] | 0.9 (0.5)  [-0.2 – 1.9] | 0.91 |
| ΔD_50_ | 0.1 (0.2)  [-0.3 – 0.4] | 0.4 (0.6)  [-0.3 – 1.4] | 0.0002 | 0.5 (0.5)  [-0.5 – 1.3] | 0.9 (0.6)  [-0.1 – 2.2] | 0.44 |
| ΔD_95_ | 0.0 (0.3)  [-0.9 – 0.6] | 0.6 (0.6)  [-0.6 – 1.9] | <0.0001 | 1.1 (3.6)  [-3.3 – 16.3] | 1.5 (1.6)  [-1.0 – 5.3] | 0.52 |
| ΔD_98_ | -0.2 (0.7)  [-2.9 – 0.7] | 0.6 (1.0)  [-2.5 – 2.6] | 0.0027 | 0.8 (2.07)  [-2.4 – 7.1] | 1.7 (2.3)  [-2.7 – 7.6] | 0.59 |
| ΔD_mean_ | 0.1 (0.2)  [-0.3 – 0.4] | 0.5 (0.4)  [-0.3 – 1.3] | <0.0001 | 0.6 (0.5)  [-0.4 – 2.0] | 1.0 (0.5)  [0.0 – 2.1] | 0.40 |

**Appendix Table 3.** Gamma analysis results for glioma and brain metastasis patient subgroups. (DTA: dose and distance-to-agreement, SD: standard deviation)

| Group | Glioma | Metastasis |
| --- | --- | --- |
| Dose and DTA criterion | **Mean pass rate (SD) [Range] [%]** | |
| 1%/1 mm [%] | 82.1 (7.6)  [65.5 – 95.6] | 95.2 (8.5)  [68.6 – 100.0] |
| 2%/2 mm [%] | 98.0 (2.1)  [89.7 – 99.8] | 99.2 (2.0)  [91.0 – 100.0] |
| 3%/3 mm [%] | 99.7 (0.5)  [97.4 – 100.0] | 99.8 (0.4)  [98.1 – 100.0] |

**Appendix Table 4.** Patient positioning accuracy results with absolute error values in each coordinate direction for CBCT. Total translation error is defined as the vector sum of translation in three primary coordinate directions. (AP: anterior-posterior, CC: cranio-caudal, LR: left-right, SD: standard deviation)

| Group | Glioma | Metastasis |  |
| --- | --- | --- | --- |
| Translation | **Difference (SD) [Range] [mm]** | | **p** |
| LR | 0.5 (0.5)  [0.0 – 1.7] | 0.6 (0.6)  [0.0 – 1.8] | 1.0 |
| AP | 0.4 (0.2)  [0.1 – 0.7] | 0.8 (0.8)  [0.0 – 2.5] | 0.32 |
| CC | 0.6 (0.6)  [0.1 – 1.9] | 0.6 (0.5)  [0.0 – 1.1] | 0.65 |
| Total translation | 0.9 (0.6)  [0.3 – 2.6] | 1.4 (0.9)  [0.5 – 3.3] | 0.24 |
| Rotation | **Difference (SD) [Range] [˚]** | | **p** |
| Pitch | 0.1 (0.1)  [0.0 – 0.3] | 0.1 (0.1)  [0.0 – 0.2] | 1.0 |
| Roll | 0.1 (0.1)  [0.0 – 0.3] | 0.05 (0.05)  [0.0 – 0.2] | 0.03 |
| Yaw | 0.1 (0.1)  [0.0 – 0.3] | 0.1 (0.1)  [0.0 – 0.2] | 0.84 |


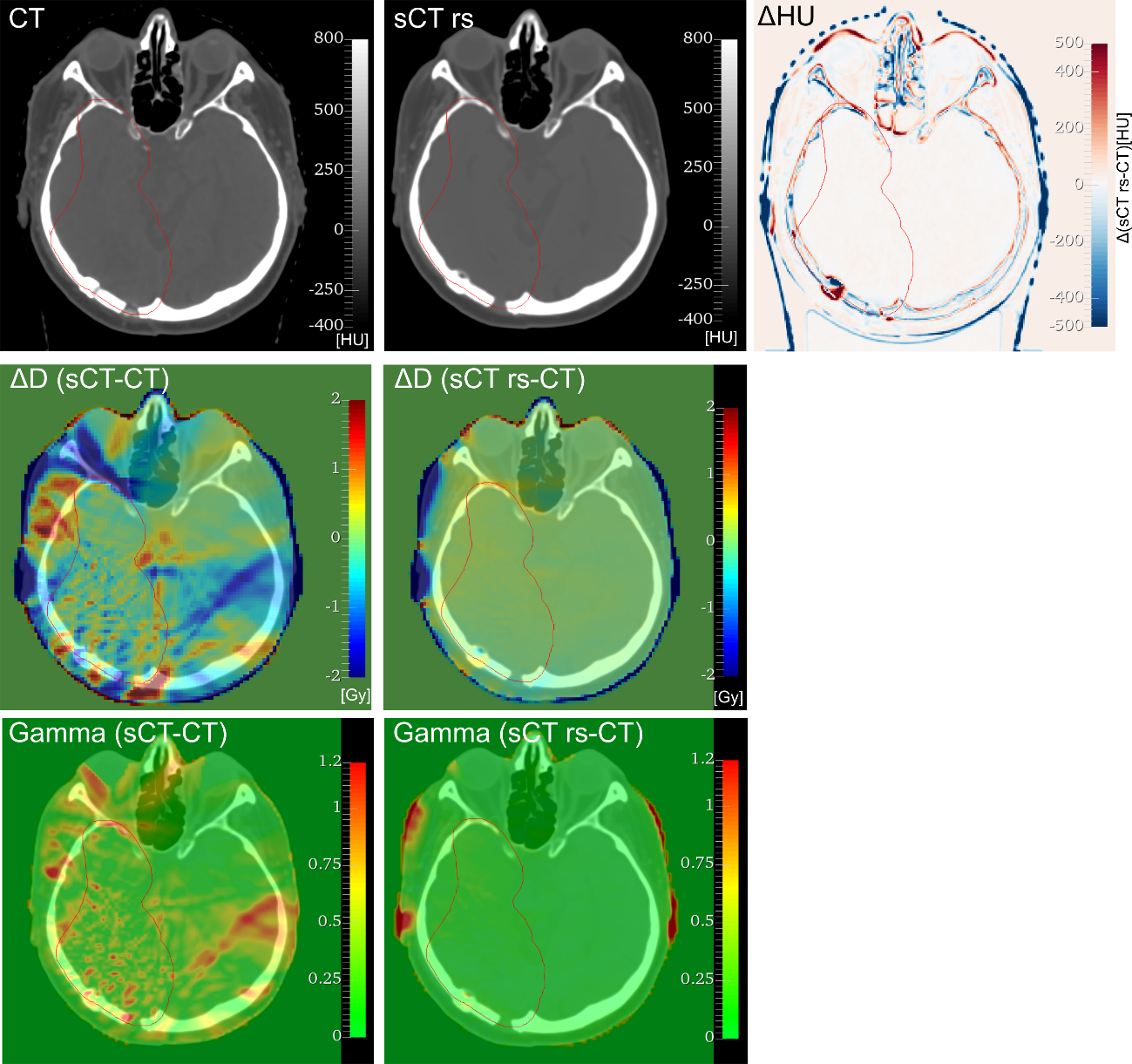
**Appendix Figure 2.** An overview of the effects of registration and resampling for the outlier glioma patient. Top row: CT and resampled sCT images, and corresponding HU difference map. Middle row: Absolute dose differences between the non-resampled sCT and CT plans (left) and the same difference between the resampled sCT and sCT plans (right). Bottom row: The gamma maps for the non-resampled sCT and CT plans (left) and the corresponding gamma map for the resampled sCT and CT plans (right). (CT: computed tomography, sCT: synthetic computed tomography, rs; resampled, HU: Hounsfield unit, D: dose)


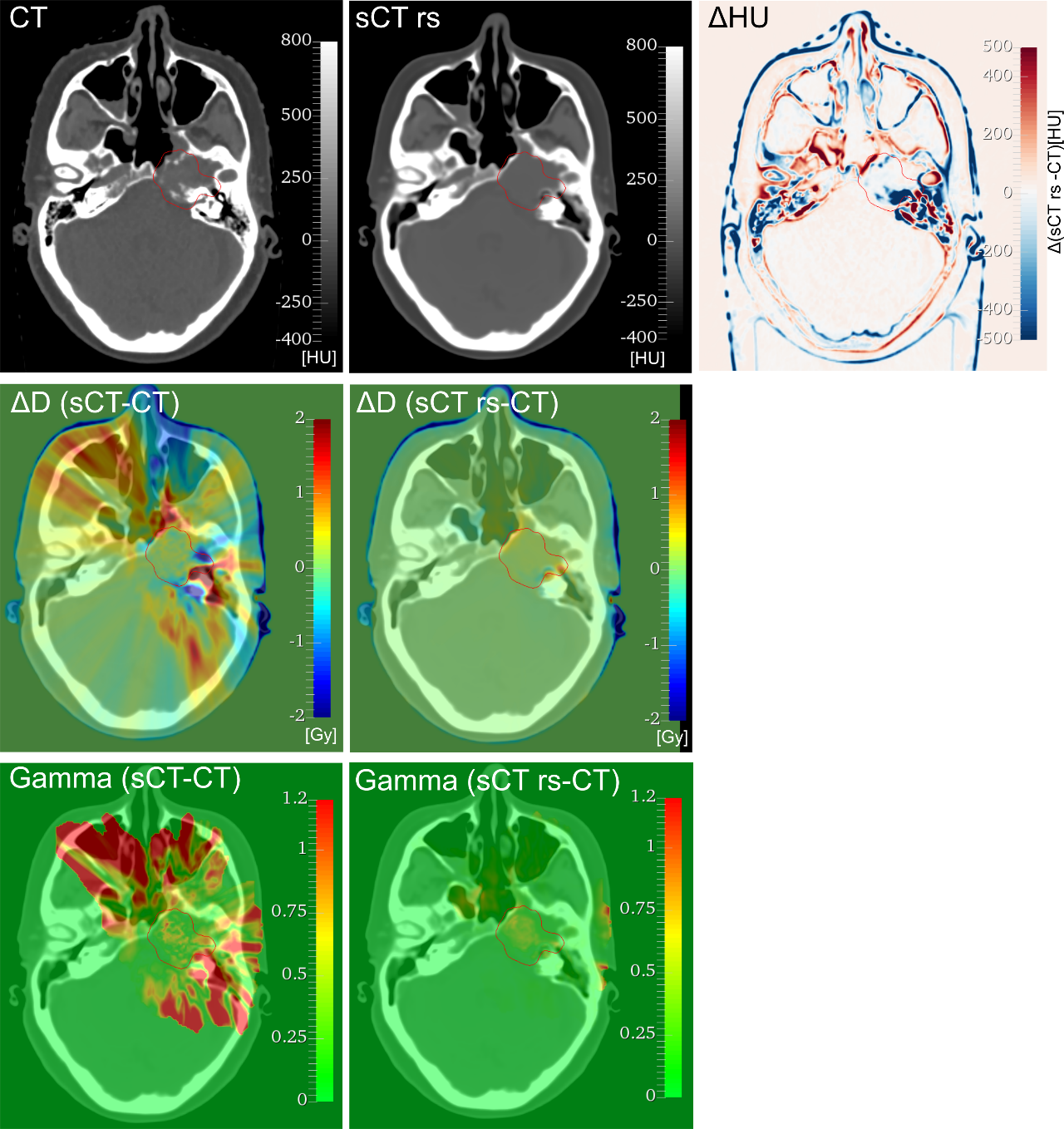
**Appendix Figure 3.** An overview of the effects of registration and resampling for the outlier metastasis patient. Top row: CT and resampled sCT images, and corresponding HU difference map. Middle row: Absolute dose differences between the non-resampled sCT and CT plans (left) and the same difference between the resampled sCT and sCT plans (right). Bottom row: The gamma maps for the non-resampled sCT and CT plans (left) and the corresponding gamma map for the resampled sCT and CT plans (right). (CT: computed tomography, sCT: synthetic computed tomography, rs; resampled, HU: Hounsfield unit, D: dose)
